# Development of South Australian-Victorian Prostate Cancer Health Outcomes Research Dataset

**DOI:** 10.1186/s13104-016-1855-3

**Published:** 2016-01-22

**Authors:** Rasa Ruseckaite, Kerri Beckmann, Michael O’Callaghan, David Roder, Kim Moretti, John Zalcberg, Jeremy Millar, Sue Evans

**Affiliations:** Department of Epidemiology and Preventive Medicine, Monash University, Melbourne, VIC Australia; Centre for Population Health, Sansom Institute for Health Research, University of South Australia, Adelaide, SA Australia; South Australian Prostate Cancer Clinical Outcomes Collaborative, Department of Urology, Repatriation General Hospital, Adelaide, SA Australia; Flinders Centre for Innovation in Cancer, Flinders University, Adelaide, SA Australia; Freemasons Foundation Centre for Men’s Health and Discipline of Medicine, University of Adelaide, Adelaide, SA Australia; William Buckland Radiation Oncology Department, the Alfred, Melbourne, VIC Australia

**Keywords:** Prostate cancer, Clinical registry, Patterns of care, South Australian-Victorian Prostate Cancer Health Outcomes Research Data

## Abstract

**Background:**

Prostate cancer is the most commonly diagnosed and prevalent malignancy reported to Australian cancer registries, with numerous studies from single institutions summarizing patient outcomes at individual hospitals or States. In order to provide an overview of patterns of care of men with prostate cancer across multiple institutions in Australia, a specialized dataset was developed. This dataset, containing amalgamated data from South Australian and Victorian prostate cancer registries, is called the South Australian-Victorian Prostate Cancer Health Outcomes Research Dataset (SA-VIC PCHORD).

**Results:**

A total of 13,598 de-identified records of men with prostate cancer diagnosed and consented between 2008 and 2013 in South Australia and Victoria were merged into the SA-VIC PCHORD. SA-VIC PCHORD contains detailed information about socio-demographic, diagnostic and treatment characteristics of patients with prostate cancer in South Australia and Victoria. Data from individual registries are available to researchers and can be accessed under individual data access policies in each State.

**Conclusions:**

The SA-VIC PCHORD will be used for numerous studies summarizing trends in diagnostic characteristics, survival and patterns of care in men with prostate cancer in Victoria and South Australia. It is expected that in the future the SA-VIC PCHORD will become a principal component of the recently developed bi-national Australian and New Zealand Prostate Cancer Outcomes Registry to collect and report patterns of care and standardised patient reported outcome measures of men nation-wide in Australia and New Zealand.

## Findings

### Background

Clinical quality registries have been established in various healthcare settings to capture and provide measurement and longitudinal benchmarking of patients’ demographic, clinical and quality of life (QOL) data. The main function of these registries is to deliver this information and feedback to healthcare providers for quality management and to implement practice changes of clinical and economic significance [1–3]. Many registries serve as tools for clinical trials in monitoring drug safety and efficacy and cost-effectiveness for various conditions, including surgical, neurological, renal, rheumatology, gynaecology, heart, trauma and others [4–7]. Hundreds of research reports and publications arising from studying clinical registry data in the US, Europe, some Asian countries as well as in Australia can be found in the scientific literature [1, 2, 8–14].

Cancer is one of the most frequent health conditions where clinical quality registries are widely used to inform, monitor and evaluate trends in incidence, survival and management of health services and patient outcomes [15–19]. Prostate cancer is the most commonly diagnosed and prevalent malignancy reported to Australian cancer registries and globally, with numerous studies using registry data and sampling to report on patterns of care, treatment options and long-term survival [20–26].

While long term survival following a diagnosis of prostate cancer is relatively good (~92 % at 5 years) [27], there is considerable morbidity associated with the treatment and management of prostate cancer. Currently little research has examined the effectiveness of various treatment pathways within the Australian setting. Much of the evidence that guides clinical management decisions in Australia has been derived from international studies. It is unclear whether clinical characteristics, treatment patterns and outcomes among Australian men are comparable with those of men in the USA or Europe, where much of the international research is based [21, 28].

To address these questions numerous studies have been conducted using data from prostate cancer registries in South Australia and Victoria in Australia. However, these studies have been limited to hospital groups within single states and have not examined patterns across multiple jurisdictions [20, 21, 28, 29]. Studies across multiple jurisdictions and multiple registries would generally provide broader coverage and strengthen the evidence base for evaluating patterns of care and patient outcomes; and point to opportunities for improving health outcomes of men with prostate cancer in Australia. Results from multiple institutions would generally be more powerful than those from single jurisdictions as they would be well placed to find and control for additional sources of variation and take advantage of natural policy experiments [30–32].

Victoria and South Australia represent 32 % of the Australian population. Both states have distinct demographic profiles, which collectively reflect the population distribution across Australia. Most of South Australia’s population (85 %) resides in the inner or outer urban areas of the capital city, with a small proportion residing in remote locations that are quite distant from health care services, similar to the states of Western Australia and the Northern Territory [33]. In these states, tertiary care is centralised in the capital cities; hence radical treatment for prostate cancer is generally only available in metropolitan hospitals. In contrast, a larger proportion of the population of Vic reside in major centres outside the capital city, and tertiary care tends to be more decentralised, similar to other eastern states. While geographical access to health care services differs across states, particularly access to tertiary hospitals, universal health care (including free hospital care) is available to all Australian residents.

The main objective of this study was to develop a South Australian-Victorian Prostate Cancer Health Outcomes Research Dataset (SA-VIC PCHORD) in order to provide an overview of socio-demographic and clinical characteristics as well as treatment patterns and outcomes of prostate cancer patients across two Australian states. This paper will present a technical description of SA-VIC PCHORD, containing merged records from two clinical registries in the states of SA and Vic, Australia. These states were selected for this study as they are currently the only two established longitudinal, third-party collected prostate cancer registries in this country.

## Methods

### Prostate Cancer Health Outcomes Research Unit (PCHORU)

In 2013, the Movember Foundation, a Men’s Health Charity Organisation, funded an initiative to seek consensus on implementation of the Australian and New Zealand Prostate Cancer Outcomes Registry (PCOR–ANZ) [14]. Subsequently, research collaboration has been established between the University of South Australia, Monash University, the Movember group and the South Australian Health and Medical Research Institute (SAHMRI) to establish the Movember Prostate Cancer Health Outcomes Research Unit (PCHORU). Unit aims include: (1) investigating risk adjusted treatment outcomes and care patterns for Australian men with prostate cancer, (2) assessing quality and appropriateness of care, (3) comparing care patterns and outcomes of prostate cancer with international benchmarks, (4) investigating socio-demographic inequalities in cancer treatment, (5) investigating survivorship and quality of life of men with prostate cancer, (6) developing improved methods of risk stratification for prostate cancer, (7) developing an improved composite outcome indicator of effectiveness of prostate cancer care, (8) and undertaking other research directed at improving outcomes of prostate cancer for men and their partners. To address these aims, the Unit will conduct a series of studies, using retrospective data from the South Australian and Victorian prostate cancer registries. Most importantly, based on results, action plans will be developed to increase the quality and effectiveness of prostate cancer care in Australia.

### Data sources

To address PCHORU goals, we developed a dataset, containing amalgamated records of men with prostate cancer from South Australia and Victorian prostate cancer registries.

All men, above 18 years of age, who have been diagnosed or treated for prostate cancer in participating sites in SA or Vic, are eligible to participate in the registries. The method of identifying eligible patients is dependent on the source of prostate cancer notifications. Notifications may come from individual hospitals, surgical centres, or pathology providers. Patient information and consent forms, explaining the registry, data collection, and option to opt-out at any time, are provided to all prospective participants. A waiver of consent enables collection of diagnostic and treatment data on men who have died before providing consent, and on men diagnosed via a transurethral resection of the prostate (TURP), for whom consent must be obtained from their treating doctor [14].

The Victorian Prostate Cancer Registry (now termed the Prostate Cancer Outcomes Registry-Victoria, or PCOR-Vic), based at Monash University, was established in 2008 [34]. The registry collects data on prostate cancer cases from 38 metropolitan and regional public and private hospitals in Vic. Based on the latest update from Victorian Cancer Registry [35], these sites account for about 70 % of incidence prostate cancer cases in that State, and more than 10,000 men have been accrued. More information detailing patients’ recruitment and methods for data collection, can be found elsewhere [28].

The South Australian Prostate Cancer Clinical Outcomes Collaborative (SA-PCCOC) database, based in South Australia, was established in 1998 to include men with prostate cancer at three major teaching and treatment hospitals in South Australia [36]. The database has been expanded more recently to include private treatment facilities. Currently the registry contains data on more than 10,000 patients. Approximately 75 % of the urologists practicing in South Australia are actively engaged in recruiting participants and contributing data to SA-PCCOC. Coverage includes patients treated at all public hospitals and most private practices, including public and private radiotherapy services. Details of the registry, including methods for data collection, are described elsewhere [20].

Both registries contain data on patient demographic characteristics, initial diagnosis and disease staging information, prostate specific antigen (PSA) history, clinical examination results, treatment details, comorbidities and complications. Follow-up data are derived from the monitoring of PSA values, clinical evidence of recurrence, any further biopsy and pathology reported, date and cause of death as well as patient reported symptoms and patient reported QOL data.

### Ethics and permissions

Numerous meetings were held amongst PCHORU steering committee members, registry managers and researchers to determine what data items should be included in the SA-VIC PCHORD. A protocol describing inclusion, exclusion criteria, time frames and data items was then developed and human research ethics approvals for the project were obtained from Monash University and the University of South Australia. Data request applications detailing data items to be extracted were submitted to each of the registries and the necessary data were extracted, de-identified and cleaned. Data extracted from the SA-PCCOC registry were sent to Monash University researchers, where it was merged with the PCOR-Vic data.

### Data storage

The amalgamated dataset (SA-VIC PCHORD) is password protected and housed on a secure network at Monash University Department of Epidemiology and Preventive Medicine, Melbourne, Victoria. Monash Registry Database security is maintained using encryption of data, a managed and audited protocol for access, training and accreditation of personnel, role-based access and authentication of data. Monash Registry Databases are housed and managed in an ISO 27001 certified environment. The ISO 27001 certification incorporates the Privacy Act (1988) and Health Records Act (2001) within its applicability statement. [28].

A copy of the amalgamated dataset was sent to the SA-PCOCC data custodian and is also stored on a secure password protected computer at The University of South Australia (School of Population Health, SAHMRI, Adelaide, South Australia). Access to the dataset is limited to the investigators of this study.

## Data items included in SA-VIC PCHORD

### Patient characteristics

A total of 13,598 records of prostate cancer men diagnosed and consented between 2008 and 2013 in South Australia and Victoria were merged into the SA-VIC PCHORD. Data categories, individual variables, value labels and completeness of the SA-VIC PCHORD data are presented in Table 1.

**Table 1:** A detailed description of SA-PCHORD variables according to the information categories, type and completeness of the data

Information category	Existing variable	New variable	Value label	Variable type	Value categories	Completeness (%)
Patient	Patient identification number		Randomly generated patient identification number	Numeric		100
	State		State identification	Categorical	[VIC; SA]	100
		Age at diagnosis	Patient’s age at diagnosis, derived from date of birth	Numeric		100
		Age groups	Ten year age groups, derived from age at diagnosis	Categorical	[<55; 55–65; 66–75; >75]	100
	Date of death		Date of death	Date		NA
	Postcode of residence		Postcode of residence	Numeric		97.6
		SEIFA score	Postcode of residence linked with SEIFA-2011	Numeric		97.6
		SEIFA decile	Postcode of Residence linked with SEIFA-2011	Categorical	[1..10]	97.6
Diagnosis	Date of diagnosis		Date of diagnosis	Date		100
		Year of diagnosis	Derived from date of diagnosis	Numeric		100
	Diagnosis procedure type		Biopsy type	Categorical	[TRUS; TURP; other]	96.9
	PSA at diagnosis		PSA levels at diagnosis	Numeric		86.6
	Gleason primary score		Biopsy Gleason primary score at diagnosis	Numeric		92.9
	Gleason secondary score		Biopsy Gleason secondary score at diagnosis	Numeric		92.8
	Gleason tertiary score		Biopsy Gleason tertiary score at diagnosis	Numeric		89.6
	Gleason total score		Biopsy Gleason total score at diagnosis	Numeric		97.5
	Biopsy number		Biopsy number to identify multiple biopsies	Numeric		100
	Biopsy type		Biopsy type	Categorical	[TRUS; TURP; TURBT; transperineal biopsy; clinical investigation (e.g. bone scan, CT, MRI); histology of metastatic site (bone biopsy or lymph node biopsy); prostatectomy (simple, diagnostic); other; unknown]	97.4
	Biopsy date		Biopsy date	Date		100
	Total number of cores		Total number of cores taken	Numeric		78.1
	Total number of positive cores		Total number of positive cores	Numeric		78.1
	Gleason primary score		Biopsy Gleason primary score for each biopsy	Numeric		89.7
	Gleason secondary score		Biopsy Gleason secondary score for each biopsy	Numeric		89.5
	Gleason tertiary score		Biopsy Gleason tertiary score for each biopsy	Numeric		NA
	Gleason total score		Biopsy Gleason total score for each biopsy	Numeric		93.8
	Perineural invasion		Perineural invasion flag	Categorical	[Yes; no]	100
	Lymphovascular invasion		Lymphovascular invasion flag	Categorical	[Yes; no]	100
	T-stage at diagnosis		T-stage at diagnosis	Categorical	[TX (cannot be assessed);T0; T1; T1a; T1b; T1c; T2 l; T2a; T2b; T2c; T3; T3a; T3b; T4; T4a; T4b]	53.4
	N-stage at diagnosis		N-stage at diagnosis	Categorical	[NX (cannot be assessed); N0; N1; inconclusive]	63.7
	M-stage at diagnosis		M-stage at diagnosis	Categorical	[MX (cannot be assessed); M0; M1a; M1b; M1c; inconclusive]	63.7
		NCCN	NCCN stage calculated according to the formula from clinical T-stage, PSA level and Gleason score at diagnosis	Categorical	[Low; intermediate; high; very high; metastatic]	92.4
Treatment	Treatment type		First treatment type	Categorical	[AA; ADT; Brachytherapy; Chemotherapy; EBRT; HDR; LDR; RALRP; low dose agonists; ORPR other; recent biopsy; WW]	93
	Treatment date		First treatment date	Date		93
		Treatment group	Treatment group derived from treatment type	Categorical	[Surgery; radiation therapy; ADT; AS, WW; other; unknown]	92.8
		Time to the first treatment	Time (days) to the first treatment, derived from date of diagnosis and date of first treatment	Numeric		72.5
Radiotherapy						N = 38,052
	Radiotherapy type		Type of radiotherapy received, can be multiple treatments	Categorical	[EBRT radical; EBRT palliative; brachytherapy]	(100)
	Start radiotherapy date		Date of start radiotherapy	Date		100
	Dose		Dose (amount) of radiotherapy	Numeric		32.9
	Fractions		Fractions of radiotherapy given	Numeric		29.7
	Dose rate		Type of dose of radiotherapy	Categorical	[LDR; HDR]	21.7
Surgery						N = 5874
	Operation date		Date of operation	Date		
	Operation type		Type of the operation	Categorical	[RALRP; laparoscopic radical; ORPR; other]	(97.8)
	Gleason primary score		Gleason primary score at operation	Numeric		96.4
	Gleason secondary score		Gleason secondary score at operation	Numeric		96.4
	Gleason tertiary score		Gleason tertiary score at operation	Numeric		10.5
	Gleason total score		Gleason total score at operation	Numeric		96.4
	Extraprostatic invasion		Extraprostatic invasion flag	Categorical	[Yes; no]	97.7
	Perineural invasion		Perineural invasion flag	Categorical	[Yes; no]	97.7
	Lymphovascular invasion		Lymphovascular invasion flag	Categorical	[Yes; no]	99.1
	Post- surgery pathology T		Post-surgery pathology T stage	Categorical	[TX (cannot be assessed);T0; T1; T1a; T1b; T1c; T2 l; T2a; T2b; T2c; T3; T3a; T3b; T4; T4a; T4b]- T4b	97
	Post- surgery pathology N		Post-surgery pathology N stage	Categorical	[NX (cannot be assessed); N0; N1; inconclusive]	96
	Seminal vesical invasion		Seminal vesical invasion flag	Categorical	[Yes; No]	95.7
	Margin status		Pathological margins status	Categorical	[Present; absent; abuts margin; equivocal]	96.5
ADT	Type of treatment		Type of hormones prescribed	Categorical	[Chemical; surgical]	N = 2734 (99.7)
	Start date		Start date of hormones treatment	Date		100
	Class		Type/class of hormones	Categorical	[Palliative; adjuvant]	100
Chemotherapy	Chemo agent		Chemotherapy treatment flag	Categorical	[Yes; no]	N = 70 (100)
	Chemo start date		Start of chemotherapy treatment	Date		100
Other treatment	Other treatment type			Categorical	[AS; WW; No treatment]	N = 2602 (100)
	Other treatment date			Date		15 (VIC–NA)
PSA			Other treatment type			
	Additional/subsequent/secondary treatment type		Date of other/additional treatment	Categorical	[AA; ADT; Brachytherapy; chemotherapy; EBRT; HDR; LDR; RALRP; low dose agonists; ORRP; other; recent biopsy; WW]	N = 12,039 (100)
		Treatment group	Treatment group derived from treatment type	Categorical	[Surgery; radiation therapy; hormones; AS/WW; other; unknown]	100
	Treatment date		Treatment date	Date		100
	PSA available		PSA available flag	Categorical	[Yes; no]	100
	PSA date		Date of PSA taken	Date		95.3
	PSA value		PSA value at treatment	Numeric		94.5

Both the PCOR-Vic and SA-PCCOC registries collect identifiable patient, clinician and treating institution information. To avoid identical entries, we checked patients’ name, surname, date of birth, date of diagnosis and clinician information. No identical entries were found in both registries. To protect privacy, and before merging data from the two registries, all identifiable data, such as names, patients’ dates of birth, residential addresses, clinicians’ surnames and hospital names were removed, and randomly created numbers (Patient Ids) were assigned to each case of the merged set. South Australia and Victoria are neighbouring states; therefore it is possible that some patients seek care in the alternate state. A State variable, based on the origin of the diagnosing institution, has been created to identify patients in both states, and it is assigned to each patient after his details have been entered to the registry; thus ensuring that no duplicated records occur.

After calculating age at the diagnosis, patient’s date of birth was deleted from the merged file. The age group variable was also created according Australian Bureau of Statistics (ABS) [37] 5 year age categories. A linkage with socio-economic indexes for areas (SEIFA) [38] on the basis of the patient residential postcode was performed and two additional data items (SEIFA decile and score) were created. SEIFA is a multidimensional area level measure of socioeconomic advantage and disadvantage based on characteristics of one neighbourhood relative to others. SEIFA was developed and validated (and regularly updated) by ABS using census data for collector districts and it is commonly used in Australian research studies. The SEIFA index is a composite of a number of average measures within an area including equivalent household income, occupancy type, level of educational attainment, level of employment/unemployment, occupational skill level, crowding, car ownership, marital status, housing and income support.

Date of death was available in both registries and was included into the merged dataset. In South Australia, vital status and cause of death (prostate cancer or other) is assessed through linkage with the state death registry, managed by the Office of Births Deaths and Marriages, and is updated regularly for individual cases where death is reported in the case notes or National Death Index. In Victoria, death information is received via data linkage with the Victorian Cancer Registry (which in turn periodically links to the Victorian Registry of Births, Deaths and Marriages). In both cases, vital status was updated immediately prior to merging the data extracts from each state. Vital status, as well as the other data in the registry, is regularly updated in each State, and the updated information is fed onto the SA-VIC PCHORD dataset on annual basis.

### Diagnostic characteristics

Diagnostic information included date of diagnosis, type of diagnostic procedures, PSA levels, Gleason scores and clinical staging data. Type of diagnostic procedure was available for 96.9 % of patients and originally grouped into the following categories: Trans-rectal ultrasound (TRUS), transurethral resection of prostate (TURP), transperineal biopsy, clinical investigation methods such as computer tomography (CT), magnetic resonance (MRI), histology or others. Since the vast majority of patients were diagnosed by TURP or TRUS, these categories were re-coded into a new variable, consisting of three groups: (1) TRUS, (2) TURP, and (3) Other methods (“other” accounting for 3.1 % of patients).

PSA level at diagnosis was available for 86.6 % patients. For research purposes this variable was grouped into the four categories: (1) <4 ng/mL, (2) 4–10 ng/mL, (3) 10–20 ng/mL and (4) >20 ng/mL. Missing PSA levels were classified as “unknown”. Primary, secondary, tertiary and total Gleason scores were also included in the dataset and grouped into the following grade groups: (1) total Gleason score ≤6, (2) Gleason score 3 + 4, (3) Gleason score 4 + 3, (4) Gleason score 8, and (5) Gleason score ≥9. Total Gleason score was available for 97.5 % of cases. Missing Gleason scores were classified as “unknown”. The boundaries (cut-points) of Gleason score categories were chosen based on a recently published contemporary prostate cancer grading system [39].

Clinical T-category, N-category and M-category at diagnosis is based on the size and/or extent (reach) of the primary tumour (T), the amount of spread to nearby lymph nodes (N), and the presence of distant metastasis (M) or secondary tumours formed by the spread of cancer cells to other parts of the body [40]. Clinical T- category was recorded for 53.4 %, N- and M- categories—for 63.7 % cases. The National Comprehensive Cancer Network (NCCN) risk criteria for disease progression were used to classify patients into low-, intermediate-, high- and very high/metastatic risk disease [41]. Where the clinical T category was not recorded, the patient was categorized as at low risk of disease progression if the Gleason score was ≤6 and the PSA concentration was <10 ng/mL [21]. For those patients, whose PSA concentration and/or Gleason score was not available, but their clinical staging category was “T3b-T4” or “Any T, N1 or Any T, Any N, M1”, the risk of the disease was assigned to very high/metastatic. NCCN classification was calculated for 92.4 % cases. Information on any subsequent biopsies was also available in both datasets and it was merged into SA-VIC PCHORD (Table 1).

### Treatment characteristics

The first treatment type, which patients received and the date of treatment was recorded for 93 % of patients. Treatment types were coded differently in South Australian and Victorian source registries. However six major treatment categories could be used: (1) surgery (prostatectomy), (2) radiotherapy, (3) androgen deprivation therapy (ADT), (4) active surveillance (AS), (5) watchful waiting (WW) and (6) others (i.e. high intensity focused ultrasound, chemotherapy etc.). For those patients whose treatment information was not recorded, treatment type was coded as “unknown”. Time to the treatment was calculated as difference (in days) between dates of diagnosis and first treatment. Distance to treatment centre (in kilometres) was computed using geocoded residential and hospital information. [42]. All treating hospitals in South Australia and 71 % of the hospitals in Victoria were metropolitan.

Information on any subsequent treatment was also available and was included in the SA-VIC PCHORD. Radiotherapy information included the type of the treatment (i.e. radical, palliative or brachytherapy), start date, dose, fractions and dose rate (low or high). Surgery information included operation date, type, surgical pathology Gleason scores, positive surgical margins and pathological TNM categories. Hormone and chemotherapy treatment details were also available for those who undertook these treatments and data included type and commencement date. AS and WW were distinguished from each other as separate options as recorded in each registry.

### Post-diagnostic PSA measures

Both states’ registries record post-diagnostic PSA levels. In Victoria, PSA at the time of each new treatment was recorded to 24 months post-diagnosis, while in South Australia, all PSA measures were captured irrespective of time point. For the SA-VIC PCHORD, data were combined to include PSA level at or immediately prior to treatment. For South Australia, PSA measured at the closest date before each treatment was assigned as the ‘treatment PSA’. Only PSA measures for treatment within 2 years of diagnosis were included to be consistent with Victorian data. PSA levels at 12 and 24 months post diagnosis follow-up were also recorded in the combined data set. For South Australia, PSA levels recorded within 1 month of the 12 and 24 month post-diagnosis were used for those follow-up points.

## Discussion

This project merged data from two prostate cancer registries to develop an amalgamated research dataset to describe characteristics of men with prostate cancer in South Australia and Victoria. Amalgamated datasets containing clinical information obtained from various data sources have been widely used for research purposes. A similar dataset was described by Choi et al. [43], who developed a prostate cancer research database system in Korea, incorporating information about a prostate cancer for research, including demographics, medical history, operation information, laboratory and QOL surveys from the CaPSURE database [44], the Japanese prostate cancer registry and the Center for Prostate Disease Research database in the USA. Another research dataset was described by Pathy et al. [12], who merged two hospital-based breast cancer databases (University Malaya Medical Center, Malaysia, and National University Hospital, Singapore) into a regional registry of breast cancer patients diagnosed between 1990 and 2007. Methods to create clinical datasets containing linked and merged data were also summarized by Pates et al. [11] who presented their methodology and the impact of merging detailed state-wide mortality data into the master patient index tables of the clinical data repository (CDR) of the University of Virginia Health System (USA) to assist caregivers in identification of at-risk patient groups by description of those patients in the CDR who have committed suicide.

### Strengths and limitations

The main strength of the SA-VIC PCHORD is the use of clinical registry data, containing a detailed diagnosis and treatment information of the patients with prostate cancer. PCOR-Vic and SA-PCCOC enable rapid and reliable ascertainment of patterns of care relating to men diagnosed with prostate cancer and provide reports back to treating clinicians in regional and metropolitan Victoria and South Australia [28, 36]. Demographic, diagnosis and treatment data are periodically updated in both registries, and will be annually included into the SA-VIC PCHORD dataset.

However, some limitations to this study also need to be noted. Firstly, there was a variation in type and nature of certain data items in both registries as a result of which they could not be merged. For example, the type of hospital where a patient was treated in Victoria was coded as “private” or “public” depending on the hospital type; however this information was not available in South Australia. In the SA-PCCOC database, patients are classified as being either ‘public’ or ‘private’ rather than classifying health care facilities.

Another issue relates to missing data: for example, PSA level at diagnosis was missing for 13.4 % of cases and clinical T- stage was not available for nearly half of patients, which was crucial when calculating NCCN staging [45]. A similar problem occurred with treatment information. Treatment type was not recorded for 7 % of cases, which made it difficult to interpret whether these patients did not have any treatment, or were under active surveillance or WW.

Quality of life data from the two datasets could not be merged due to systematic differences in administration methods, tools used and data collection time points. At 12 and 24 months after the date of diagnosis, participants in Victoria were contacted by telephone to verify management details and to measure general health and disease specific patient reported outcomes about their urinary, bowel and sexual function [28]. In South Australia, a disease specific tool EPIC-26 [46] is administered as a written postal survey. It is sent out to men after they are aware of their diagnosis, but before treatment commences (usually within 3 months of diagnosis), then again at 3, 6, 12, 24, 36 and 60 months post treatment.

### Future directions

It is anticipated that the dataset described in this paper will be used for numerous studies summarizing trends, survival and patterns of care in men with prostate cancer in Victoria and South Australia. The recently developed PCOR-ANZ registry will collect patterns of care and standardised patient reported QOL measures of men nation-wide in Australia and New Zealand [14]. EPIC-26 survey [46] will be administered to all the participants, including those, whose data have been already merged into SA-VIC PCHORD, prior to treatment, and at 12 months post final active treatment. This information will be incorporated into the SA-VIC PCHORD. It is envisaged that in the future, data for men with prostate cancer collected by the PCOR-ANZ registry will be incorporated into the SA-VIC PCHORD, leading to the PCHORD expanding nationwide. We expect that various data analyses will be conducted in order to assist transforming healthcare for men with prostate cancer in Australia and New Zealand in a standardised way.

## Availability and requirements

- Project name: South Australian-Victorian Prostate Cancer Health Outcomes Research Dataset.
- Project home page: http://pcr.registry.org.au/; http://www.sa-pccoc.com.
- Operating system(s): platform independent.
- Programming language: SQL.
- Other requirements: not applicable.
- License: not applicable.
- Any restrictions to use by non-academics: not applicable.

## Availability of supporting data

Data from individual registries is available under individual data access policy in each state. Access to the data is guided by strict protocols and procedures to ensure that the privacy of men and other ethical principles are maintained at all times. The data access policy and data request form for the PCOR-Vic is available to access by registering through the website address at http://www.pcr.registry.org.au/Home.aspx. Information about the SA-PCCOC data can be found at http://www.sa-pccoc.com. Requests to access data from the SA-PCCOC registry should be addressed to the research committee for review and consideration (contactus@sa-pccoc.com). The following should be addressed in each data request: analysis strategy, data availability, publication planning, ethics requirements, funding options and student supervision. The research committee review process receives input from diverse disciplines including urology, radiation oncology, medical oncology, epidemiology and data management. A data agreement will be required for each study and researchers are encouraged to provide financial support to facilitate their request. Requests for data to support commercial activities are not considered.

SA-VIC PCHORD is currently used for research purposes by the researchers listed under the institutional ethics protocols in South Australia and Victoria. Requests for SA-VIC PCHORD data can be accepted from external researchers and will be considered on individual basis. It is anticipated, that once PCOR-ANZ data is collected and included into national prostate cancer health outcomes research dataset, researchers will be able to access this data following the processes described elsewhere [14].

## Abbreviations

AA: active surveillance
ABS: Australian Bureau of Statistics
ADT: androgen deprivation therapy
CT: computed tomography
EBRT: external beam radiation
HDR: high dose radiation
LDR: low dose radiation
MRI: magnetic resonance imaging
NCCN: National Comprehensive Cancer Network
ORRP: open retropubic radical prostatectomy
PCHORU: Prostate Cancer Health Outcomes Research Unit
PCOR-ANZ: Australian and New Zealand Prostate Cancer Outcomes Registry
PCOR-Vic: Prostate Cancer Outcomes Registry—Victoria
PSA: prostate-specific antigen
QOL: quality of life
RALRP: robot assisted laparoscopic radical prostatectomy
SA: South Australia
SAHMRI: South Australian Health and Medical Research Institute
SEIFA: socio-economic index of advantage and disadvantage
SA-PCCOC: South Australian Prostate Cancer Clinical Outcomes Collaborative
SA-VIC PCHORD: South Australian-Victorian Prostate Cancer Health Outcomes Research Dataset
TURBT: transurethral resection of bladder tumour
TURP: transurethral resection of the prostate
TRUS: transrectal ultrasonography of the prostate
Vic: Victoria
WW: watchful waiting
